# Nursing Home COVID-19 Action Network: Responding With Stress First Aid and Caring for Residents With Dementia

**DOI:** 10.1093/geroni/igab046.1898

**Published:** 2021-12-17

**Authors:** Thomas Caprio, Barbara Ganzel, Adam Simning

**Affiliations:** 1 University of Rochester Medical Center, Rochester, New York, United States; 2 Ithaca College Gerontology Institute, Ithaca, New York, United States; 3 University of Rochester, Rochester, New York, United States

## Abstract

The GWEP at the University of Rochester (New York) has an established network of nursing homes participating in Project ECHO. This ECHO hub includes geriatric medicine, psychiatry, pharmacy, aging services network and the Alzheimer’s Association focusing on best practices in geriatric mental health and dementia care. With the COVID-19 pandemic, this infrastructure quickly pivoted to expansion of 80 facilities and the addition of expertise in medical direction, trauma informed care, and infectious disease. A stress first aid training module was developed in partnership with Ithaca College and the National Center for PTSD to support front line nursing home workers. Dementia care experts contributed to practical problem-solving in addressing social isolation and mental health. Work now is focusing on vaccination and how to best support trauma-informed needs of residents with dementia.

